# Adiponectin receptor PAQR-2 signaling senses low temperature to promote *C. elegans* longevity by regulating autophagy

**DOI:** 10.1038/s41467-019-10475-8

**Published:** 2019-06-13

**Authors:** Yuan-Li Chen, Jun Tao, Pei-Ji Zhao, Wei Tang, Jian-Ping Xu, Ke-Qin Zhang, Cheng-Gang Zou

**Affiliations:** 1 State key Laboratory for Conservation and Utilization of Bio-Resources in Yunnan, School of Life Sciences, Yunnan University, Kunming, Yunnan 650091 China; 2 Faculty of Basic Medicine, Kunming Medical University, Kunming, Yunnan 650031 China; 3 Department of Biology, McMaster University, Hamilton, Ontario L8S 4K1 Canada

**Keywords:** Genetics, Physiology

## Abstract

Temperature is a key factor for determining the lifespan of both poikilotherms and homeotherms. It is believed that animals live longer at lower body temperatures. However, the precise mechanism remains largely unknown. Here, we report that autophagy serves as a boost mechanism for longevity at low temperature in the nematode *Caenorhabditis elegans*. The adiponectin receptor AdipoR2 homolog PAQR-2 signaling detects temperature drop and augments the biosynthesis of two ω-6 polyunsaturated fatty acids, γ-linolenic acid and arachidonic acid. These two polyunsaturated fatty acids in turn initiate autophagy in the epidermis, delaying an age-dependent decline in collagen contents, and extending the lifespan. Our findings reveal that the adiponectin receptor PAQR-2 signaling acts as a regulator linking low temperature with autophagy to extend lifespan, and suggest that such a mechanism may be evolutionally conserved among diverse organisms.

## Abstract (web summary)

Decreased temperatures lengthen *C. elegans* lifespan by mechanisms that are not fully understood. Here the authors show that autophagy activation contributes to lifespan extension at low temperatures and that this involves adiponectin receptor PAQR-2 signaling.

## Introduction

Both environmental and genetic factors influence aging and lifespan^1,2^. For instance, mutations in several conserved genes are known to extend lifespan in the nematode *C. elegans*, including the insulin/IGF-1signalling receptor mutant *daf-2*^3^, the feeding deficient mutant *eat-2*^4^, the ubiquinone synthesis mutant *clk-1* that impairs mitochondrial respiration^5^, the Notch receptor mutant *glp-1*^6^, TOR signaling inhibition^7^, and the ribosomal S6 kinase mutant *rsks-1* that reduces translation^8,9^. Among the environmental factors, temperature can significantly impact lifespan. In both poikilotherms and homeotherms, a lower temperature is commonly associated with a longer lifespan. For example, flies and worms live longer at lower temperatures than at higher temperatures^10–12^. In mice, the reduction of core body temperature extends lifespan^13^. At present, the mechanisms by which temperature affects lifespan are not fully understood.

For *C. elegans*, the growth temperatures in laboratory conditions are typically 15–25 °C. Worms at 15–16 °C live longer than those at 25 °C^11,12,14^. One hypothesis for the longer lifespan is that low temperatures reduce metabolic rate, thereby leading to a slowed aging. However, there is increasing evidence demonstrating that genetic factors are involved in regulating lifespan in response to temperature changes^14–17^. For instance, the AFD thermosensory neurons control lifespan by regulating the DAF-12 nuclear receptor signaling at 25 °C^15^. Furthermore, Xu and colleagues showed that TRPA-1, a cold-sensitive TRP channel protein, is required for temperature reduction-mediated longer lifespan through PKC-2 (a calcium-sensitive PKC), SGK-1 (a DAF-16/FOXO kinase), and the FOXO transcription factor DAF-16 in adult worms^14,16^. A mutation in *daf-41*, which encodes the *C. elegans* homolog of p23 co-chaperone/prostaglandin E synthase-3, extends lifespan at higher temperatures and shortens lifespan at lower temperatures^17^. Together, these studies suggest that a diversity of genetic factors are associated with temperature-mediated lifespan.

Autophagy is an evolutionarily conserved process that maintains intracellular homeostasis by degrading waste cytoplasmic contents in lysosomes. It is involved in a wide variety of biological processes, ranging from development, metabolism, immunity, stress resistance, to cancer^18,19^. Beyond these aforementioned functions, autophagy is also recognized to play an essential role in the lifespan of a diversity of model organisms, such as yeast, worms, flies, and mice. For instance, mutations in autophagy-related genes lead to reduced lifespan in flies^20^ and mice^21^. In contrast, overexpression of Atg5, an autophagy-related gene essential for autophagosome formation, extends lifespan in mice^22^. Furthermore, the transcription factor HLH-30-mediated autophagy is increased and required for worms with extended lifespan in six mechanistically distinct longevity models^23^. In addition, pharmacological interventions using spermidine^24^, rapamycin^25^, and polyunsaturated fatty acids (PUFAs)^26^, provide an indication that the activation of autophagy is associated with longevity.

In this study, we investigate whether autophagy is required for long lifespan at low temperature (15 °C) in *C. elegans*. Our results demonstrate that, compared with normal temperature (20 °C), low temperature markedly induces autophagy. Knockdown of the autophagy-related genes, such as *bec-1* (the *C. elegans* ortholog of ATG6/VPS30/beclin1)^27^, *let-512* (the *C. elegans* ortholog of VPS34)^28^, and *epg-1* (the *C. elegans* ortholog of Atg13)^29^ significantly shortens the lifespan of worms at low temperature, but does not affect the lifespan of worms at normal temperature. Furthermore, we find that the induction of autophagy is required for the adiponectin receptor AdipoR2 homolog PAQR-2 signaling, a pathway for low-temperature adaptation in larva^30,31^. Two ω-6 PUFAs, γ-linolenic acid (GLA, C18:3n6) and arachidonic acid (AA, C20:4n6), are involved in the activation of autophagy at low temperature. Finally, we show that epidermal-specific autophagy is responsible for lifespan extension, which is associated with collagen maintenance at low temperature. Taken together, these observations suggest that increased autophagy in the epidermis through the adiponectin receptor PAQR-2 signaling is a mechanism for longevity at low temperature.

## Results

### Autophagy is activated at low temperature

**Fig. 1:**
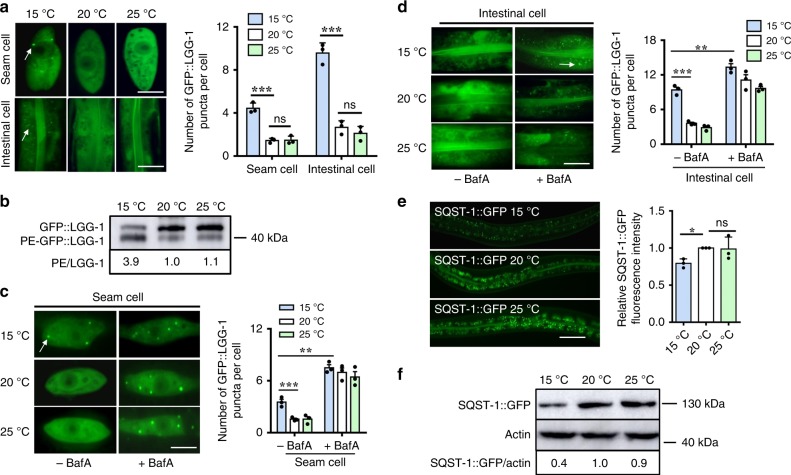
Autophagic activity is elevated at low temperature in *C. elegans*. **a** Representative images of autophagosomes (GFP::LGG-1 puncta) in the seam cells and intestinal cells of day 1 worms grown at 15, 20, and 25 °C for 24 h, respectively. The arrow denotes a representative autophagosome. The numbers of GFP::LGG-1 puncta were counted. These results are means ± SD of three independent experiments (*n* = 30–35 worms per experiment). ***P < 0.001, 15 °C versus 20 °C; ns, not significant. Scale bars: seam cells, 10 μm; intestinal cells, 20 μm. **b** The ratio of PE-GFP-LGG-1 to GFP-LGG-1 was measured by western blotting in worms. The blot shown here is typical of three independent experiments. These results are means ± SD (*n* = 3). *P* < 0.01, 15 °C versus 20 °C. **c**, **d** Quantification of GFP::LGG-1 puncta. After 1-day growth at 15, 20, and 25 °C for 24 h, adult worms were injected with 50 mM BafA or DMSO. Two hours after injection, GFP::LGG-1 puncta were quantified in seam cells (**c**) and intestinal cells (**d**), respectively. These results are means ± SD of three independent experiments (*n* = 30–35 worms per experiment). **P < 0.01; ***P < 0.001, 15 °C (+BafA) versus 15 °C (-BafA); ***P < 0.001, 15 °C (-BafA) versus 20 °C (-BafA). Scale bars: seam cells, 10 μm; intestinal cells, 20 μm. **e** The expression of SQST-1::GFP in adult worms. The right panel shows quantification of GFP levels. These results are means ± SD of three independent experiments (*n* = 50–55 worms per experiment). *P < 0.05, 15 °C versus 20 °C. Scale bars: 50 μm. **f** The levels of SQST-1::GFP were measured by western blotting. The blot shown here is typical of three independent experiments. These results are means ± SD (*n* = 3). *P* < 0.01, 15 °C versus 20 °C. *P*-values throughout were calculated using a one-way ANOVA followed by a Student-Newman-Keuls test

We first compared autophagy levels at 15 °C (low temperature), 20 °C (normal temperature), and 25 °C (high temperature), by using transgenic worms carrying GFP::LGG-1. During autophagy, LGG-1/ATG8 is sequestered at the membrane of autophagosomes and condenses into puncta, thereby reflecting the activity of autophagic processes^32^. Thus, the appearance of GFP::LGG-1-containing puncta has been demonstrated to be a reliable indicator of autophagy in worms^33^. We observed that the abundance of GFP::LGG-1-positive puncta was significantly higher in both the hypodermal seam cells and the intestine of worms at 15 °C than at 20 °C and 25 °C (Fig. 1a). Using western blotting, we detected the modification of GFP::LGG-1^34^, and found a significant increase in the ratio of phosphatidylethanolamine (PE) conjugated GFP::LGG-1 (PE-GFP::LGG-1) to nonlipidated GFP::LGG-1 in worms at low temperature (Fig. 1b). The increase in LGG-1 puncta at low temperature could result from either an induction of autophagy or a block in the turnover of LGG-1-bound autophagosomes. To distinguish between these possibilities, worms expressing GFP::LGG-1 were injected with bafilomycin A1 (BafA), an inhibitor of lysosomal acidification^35^. Worms treated with BafA showed a prominent increase in the number of GFP::LGG-1 puncta in both the seam cells and the intestine at 15 °C (Fig. 1c, d), indicating that low temperature-induced autophagic flux. To confirm this observation, we further determined the turnover of p62/SQST-1 and W07G4.5 in transgenic worms carrying either SQST-1::GFP or W07G4.5::GFP. Either SQST-1::GFP or W07G4.5::GFP was significantly decreased upon induction of autophagy in response to stimuli^33,36^. We observed a reduction in the expression of SQST-1::GFP or W07G4.5::GFP at 15 °C, compared with those at 20 and 25 °C (Fig. 1e, Supplementary Fig. 1a). Meanwhile, western blotting also revealed that the protein levels of SQST-1::GFP at 15 °C were much lower than those at 20 and 25 °C (Fig. 1f). By contrast, the mRNA levels of both *sqst-1::gfp* and *W07G4.5::gfp* were comparable in worms at 15, 20, and 25 °C (Supplementary Fig. 1b). These results indicate that autophagic activity is enhanced in worms at low temperature.

### Autophagy is involved in low temperature-induced longevity

**Fig. 2:**
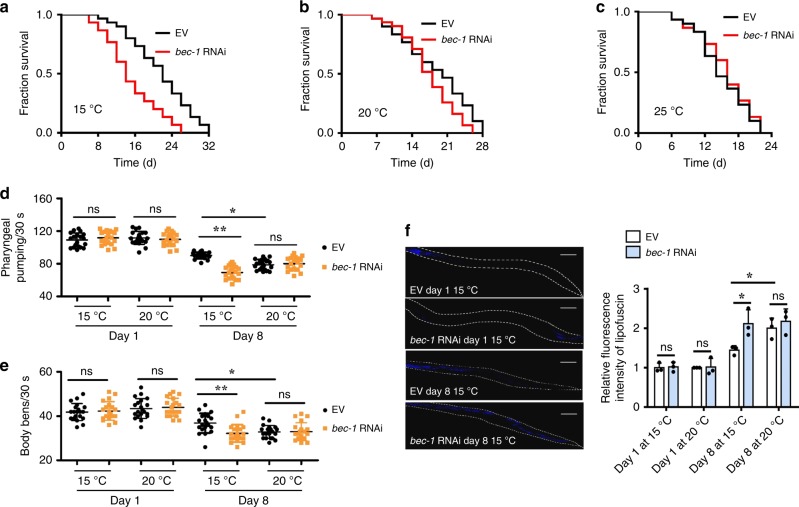
Autophagy is needed for lifespan extension at low temperature. **a**–**c** *bec-1* RNAi significantly reduced lifespan of worms at 15 °C (Log-rank test, *P* < 0.01 versus EV), but not 20 °C, and 25 °C. EV, empty vector. See Supplementary Data 1 for details. **d**, **e** Autophagy is involved in delaying the appearance of the aging markers, including pharyngeal pumping (**d**) and body bending (**e**), in worms at 15 °C, but not 20 °C. These results are means ± SD (*n* = 20). Ten worms were examined per experiment. *P < 0.05, 8-day-old worms with EV at 15 °C versus 8-day-old worms with EV at 20 °C; **P < 0.01, 8-day-old worms with EV at 15 °C versus 8-day-old worms with *bec-1* RNAi at 15 °C. ns, not significant. **f** Aging marker lipofuscin autofluorescence. These results are means ± SD of three independent experiments (*n* = 30–34 worms per experiment). *P < 0.05, 8-day-old worms with EV at 15 °C versus 8-day-old worms with EV at 20 °C, or 8-day-old worms with *bec-1* RNAi at 15 °C. ns, not significant. *P*-values (**d**–**f**) were calculated using a one-way ANOVA followed by a Student-Newman-Keuls test. Scale bars: 100 μm

As worms at low temperatures have a longer lifespan than those at warm temperatures^11,12,14^, we asked whether autophagy is required for the long lifespan at low temperature. To address this question, we suppressed autophagy by silencing *bec-1*, *let-512*, and *epg-1* by RNAi. We found that knockdown of *bec-1* prominently shortened the lifespan of worms at 15 °C (Fig. 2a, Supplementary Data 1), but not at 20 °C (Fig. 2b, Supplementary Data 1) or 25 °C (Fig. 2c, Supplementary Data 1). Similar results were obtained from wild-type worms subjected to RNAi of *let-512* (Supplementary Fig. 2a–c, Supplementary Data 1) and *epg-1* (Supplementary Fig. 2d–f, Supplementary Data 1).

Next, we determined the role of autophagy on age-associated markers, such as the pharyngeal-pumping rate, body bending, lipofuscin autofluorescence, and pharyngeal deterioration^37,38^. As expected, both the rates of pharyngeal-pumping (Fig. 2d) and body bending (Fig. 2e) were reduced in 8-day-old worms at 15 and 20 °C as compared with 1-day-old worms. Meanwhile, the accumulation of lipofuscin autofluorescence (Fig. 2f) and pharyngeal deterioration (Supplementary Fig. 3a) were increased in 8-day-old worms at 15 and 20 °C as compared with 1-day-old worms. However, the rates of pharyngeal-pumping and body bending were significantly higher, and the accumulation of lipofuscin autofluorescence and pharyngeal deterioration were significantly lower in 8-day-old worms at 15 °C than those at 20 °C. These results suggest that low temperature delays aging process. Consistent with these results, knockdown of *bec-1* by RNAi increased the prominence of ageing markers in 8-day-old worms at 15 °C (Fig. 2d–f). Similar results were obtained from worms subjected by *let-512* and *epg-1* RNAi (Supplementary Figs. 3b, c, 4a–f). Taken together, these results indicate that autophagy is involved in lifespan extension at low temperature.

Based on our observations, the 8-day-old adults at 15 °C had a higher survival rate and represented a younger stage than the 8-day-old adults at 20 °C (Supplementary Table 1). For example, for wild-type worms at 15 °C, 96.7% of the adult worms were alive on the 8th day and the 8th day represented 25% of their maximum lifespan at this temperature (Fig. 2a). In contrast, at 20 °C, 90.6% of wild-type worms were alive on the 8th day and the 8th day represented 28.6% of their maximum lifespan at this temperature (Fig. 2b). Together with previous findings^39–41^, these results indicate that the age-associated phenotypes become prominent when most worms are still alive. Thus, death is preceded by other aging phenotypes of the worm.

### The PAQR-2 signaling mediates longevity through autophagy

A previous study has shown that the TRPA-1/PKC2 signaling mediates lifespan in worms at low temperature^14^. We thus tested whether the pathway plays a role in autophagy. However, neither *trpa-1(ok999)* mutation nor *trpa-1* RNAi influenced the number of GFP::LGG-1 puncta at 15 °C (Supplementary Fig. 5a, b), implicating that this signaling pathway is unlikely involved in low temperature-induced autophagy. The transcription factor HLH-30/TFEB that regulates a set of autophagy-related genes has been shown to be required for autophagy-mediated longevity extension of *C. elegans* in the six longevity models^23^. However, we did not observe nuclear localization of HLH-30, an indicator of HLH-30 activation, in worms at 15 °C (Supplementary Fig. 6a). Moreover, knockdown of *hlh-30* by RNAi failed to influence either autophagic levels (Supplementary Fig. 6b) or the lifespan in worms at 15 °C (Supplementary Fig. 6c). Thus, there must be other mechanisms underlying low temperature-induced autophagy.

**Fig. 3:**
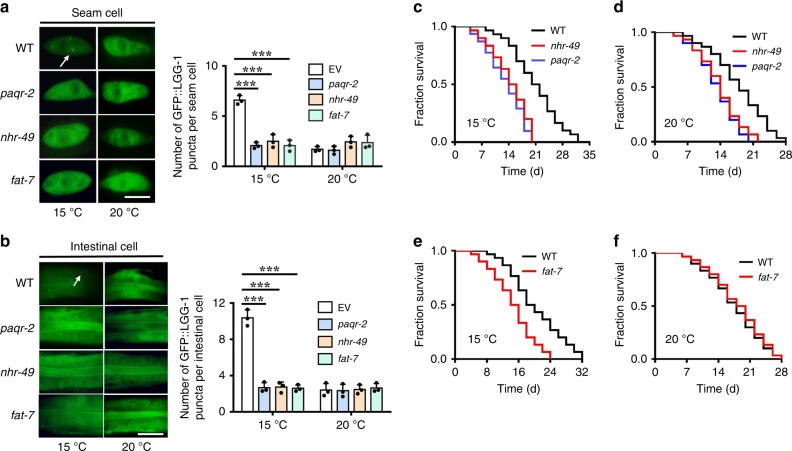
The PAQR-2 signaling acts upstream of autophagy to extend lifespan. **a**, **b** Representative images of autophagosomes (GFP::LGG-1 puncta) in the seam cells (**a**) and intestinal cells (**b**) of *paqr-2(tm3410)*, *nhr-49(nr2041)*, and *fat-7(wa36)* mutants at 15 °C and 20 °C, respectively. The arrow denotes a representative autophagosome. The numbers of GFP::LGG-1 puncta were counted in the seam cells and intestinal cells. These results are means ± SD of three independent experiments (*n* = 30–35 worms per experiment). ***P < 0.001, wild type (WT) versus mutants at 15 °C, one-way ANOVA followed by a Student-Newman-Keuls test. Scale bars: seam cells, 10 μm; intestinal cells, 20 μm. **c**, **d** Mutations in *paqr-2(tm3410)* and *nhr-49(nr2041)* shortened lifespan of worms at 15 °C (**c**) and 20 °C (**d**). *P* < 0.001, WT versus mutants at 15 °C and 20 °C, respectively. See Supplementary Data 1 for details. **e**, **f** *fat-7(wa36)* mutant worms exhibited a short lifespan at 15 °C (**e**) (*P* < 0.001, WT versus mutants), but not at 20 °C (**f**). See Supplementary Data 1 for details. *P*-values (**c**–**f**) were calculated using log-rank test

The adiponectin receptor AdipoR2 homolog PAQR-2 together with the nuclear receptor NHR-49, and the stearic CoA desaturase (SCD) FAT-7 are known to form a cold adaptation pathway in larva at low temperature^30,31^. We confirmed that low temperature upregulated the expression of *fat-7::gfp* (Supplementary Fig. 7a). In contrast, the expression of the other SCD gene *fat-6* was not altered at 15 °C (Supplementary Fig. 7b). Consistent with previous observations^31,42^, the induction of *fat-7* was dependent on PAQR-2 and NHR-49 (Supplementary Fig. 7c). We thus tested the role of this signaling pathway in autophagy. We found that mutations in *paqr-2(tm3410)*, *nhr-49(nr2041)*, and *fat-7(wa36)* caused a decrease in the abundance of GFP::LGG-1 puncta at 15 °C, but not at 20 °C (Fig. 3a, b). Similar results were obtained from worms subjected to *paqr-2* and *nhr-49* RNAi (Supplementary Fig. 8a,b). These results suggest that the adiponectin receptor PAQR-2 signaling is involved in autophagy induction at low temperature.

Next, we investigated whether this pathway is required for lifespan extension at low temperature. We found that *paqr-2(tm3410)* and *nhr-49(nr2041)* mutants had very short lifespans at both 15 °C (Fig. 3c, Supplementary Data 1) and 20 °C (Fig. 3d, Supplementary Data 1), consistent with the previous observations that *paqr-2* and *nhr-49* mutants display early lethality^30,43^. Meanwhile, knockdown of *paqr-2* and *nhr-49* by RNAi shortened the lifespan of worms at both 15 °C (Supplementary Fig. 9a, b, Supplementary Data 1) and 20 °C (Supplementary Fig. 9a, b, Supplementary Data 1). In contrast, the *fat-7(wa36)* mutants exhibited a short lifespan at 15 °C (Fig. 3e, Supplementary Data 1), but a normal lifespan at 20 °C (Fig. 3f, Supplementary Data 1). These results suggest that FAT-7 acts downstream of PAQR-2 and NHR-49 to extend lifespan at low temperature, whereas PAQR-2 and NHR-49 are required for normal lifespan.

### ω-6 PUFAs are downstream molecules of the PAQR-2 signaling

**Fig. 4:**
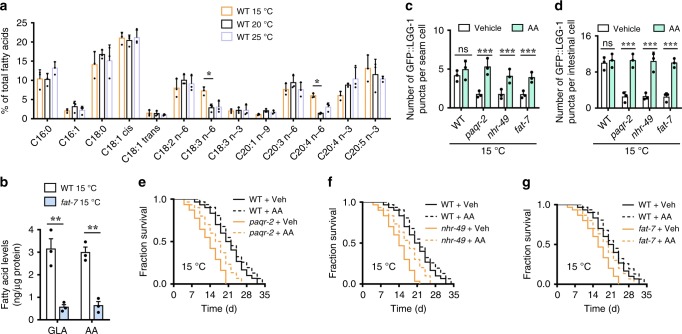
Arachidonic acid is involved in autophagy at low temperature. **a** LC-MS profiling of fatty acids in wild-type (WT) worms at 15, 20, and 25 °C. Relative content of individual fatty acid was expressed as percentage of total measured fatty acid. These results are means ± SD (*n* = 3). *P < 0.05, 15 °C versus 20 °C. **b** A mutation in *fat-7(wa36)* reduced the levels of γ-linolenic acid (GLA, C18:3n6) and arachidonic acid (AA, C20:4n6) in worms at 15 °C. These results are means ± SD (*n* = 3). **P < 0.01 versus WT. **c**, **d** Supplementation with AA (10 µM) significantly restored autophagy in both seam cells (**c**) and intestinal cells (**d**) in *paqr-2(tm3410)*, *nhr-49(nr2041)*, and *fat-7(wa36)* mutants at 15 °C. These results are means ± SD of three independent experiments (*n* = 30–35 worms per experiment). ***P < 0.001 versus vehicle. Scale bars: 100 μm. *P*-values (**a**–**d**) were calculated using a one-way ANOVA followed by a Student-Newman-Keuls test. **e**, **f** Supplementation with AA (10 µM) partially restored lifespan in *paqr-2(tm3410)* (**e**) and *nhr-49(nr2041)* (**f**) worms at 15 °C. *P* < 0.05, AA versus vehicle in *paqr-2(tm3410)* (**e**) and *nhr-49(nr2041)* (**f**) worms. Veh, vehicle. **g** Supplementation with AA (10 µM) fully rescued lifespan in *fat-7(wa36)* mutant worms at 15 °C. *P* < 0.01, AA versus vehicle in *fat-7(wa36)* worms. See Supplementary Data 1 for details. *P*-values (**e**–**g**) were calculated using log-rank test

Recently, Horvitz and colleagues revealed that PAQR-2 signaling increased intracellular levels of C11/C12 fatty acids at 15 °C^42^. At 25 °C, C11/C12 fatty acids were sequestered by an acyl-CoA dehydrogenase ACDH-11, resulting in downregulation of *fat-7*. Loss of *acdh-11* upregulated the expression of *fat-7*, leading to an increase in the levels of two ω-6 PUFAs, GLA and AA^42^. As the expression of *fat-7* was increased at 15 °C, we quantified fatty-acid content by liquid chromatography/mass spectrometry (LC/MS). We also found that the percentages of GLA and AA in wild-type worms at 15 °C were significantly higher than those at 20 and 25 °C (Fig. 4a). Mutation in *fat-7(wa36)* substantially reduced the percentages of GLA and AA at 15 °C (Fig. 4b).

Next, we tested whether these two PUFAs are involved in the PAQR-2 signaling-mediated autophagy and lifespan extension at low temperature. We found that supplementation with either AA or GLA restored the autophagy-deficient phenotype of *paqr-2(tm3410)*, *nhr-49(nr2041)*, and *fat-7(wa36)* mutants at 15 °C (Fig. 4c, d, Supplementary Fig. 10a, b). Furthermore, supplementation with one of the two PUFAs partially rescued the short lifespan in *paqr-2(tm3410)* and *nhr-49(nr2041)* mutants at 15 °C (Fig. 4e, f, Supplementary Fig. 10c, d, Supplementary Data 1), and fully restored the lifespan in *fat-7(wa36)* mutants (Fig. 4g, Supplementary Fig. 10e, Supplementary Data 1). Thus, GLA and AA function as downstream molecules of the PAQR-2 signaling to extend lifespan by activating autophagy at low temperature.

Compared with those at 20 °C, the percentages of other unsaturated fatty acids, including the monounsaturated fatty-acid oleic acid (OA, C18:1n9), two ω-6 PUFAs [linoleic acid (LA), C18:2n6], dihomo-γ-linolenic acid (DGLA, C20:3n6), two ω-3 PUFAs [ω-3 arachidonic acid (ω-3 AA), C20:4n3], and eicosapentaenoic acid (EPA, C20:5n3), were not altered at 15 °C in wild-type worms (Fig. 4a). We further tested the effect of supplementation with these additional fatty acids on autophagic activities in the *fat-7(wa36)* mutants at 15 °C. We found that supplementation with OA (100 μM), LA (200 μM), or DGLA (500 μM), but not with ω-3 AA (500 μM) or EPA (500 μM), restored the autophagy-deficient phenotype of *fat-7(wa36)* mutants at 15 °C (Supplementary Fig. 11a, b). As OA, LA, GLA, and DGLA are precursors in the biosynthesis of AA, rescue of the autophagy-deficient phenotype of *fat-7(wa36)* mutants by these four fatty acids suggested that their effects were probably due to their induced increase of AA, which in turn restored the autophagy capabilities in *fat-7(wa36)* mutants. To test this hypothesis, fatty acid composition was monitored by gas chromatography/mass spectrometry (GC/MS) after supplementation with each of the four fatty acids in *fat-7(wa36)* mutants at 15 °C. In contrast to our expectation, supplementation with any of the four fatty acids only led to an increase in the composition of the specific supplemented fatty-acid, but not in AA concentration (Supplementary Fig. 12). These results indicate that it is not only AA that promotes autophagy, but likely other fatty acids as well.

### Autophagy in the epidermis is required for longevity

**Fig. 5:**
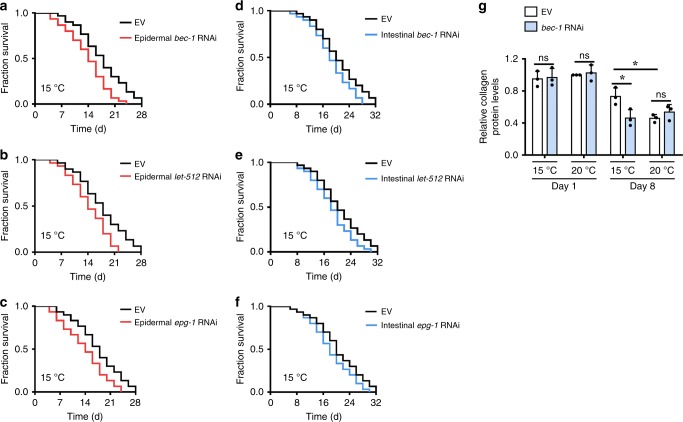
Autophagy in the epidermis is involved in lifespan extension at low temperature. **a**–**c** Epidermal-specific knockdown of *bec-1* (**a**), *let-512* (**b**), and *epg-1* (**c**) by RNAi significantly reduced the lifespan of worms at 15 °C. *P* < 0.01 versus EV. **d**–**f** Intestinal-specific knockdown of *bec-1* (**d**), *let-512* (**e**), and *epg-1* (**f**) by RNAi did not influence the lifespan of worms at 15 °C. *P* > 0.05 versus EV. *P*-values (**a**–**f**) were calculated using log-rank test. See Supplementary Data 1 for details. **g** The relative collagen levels in 8-day-old worms at 15 °C were markedly higher than those at 20 °C. Inhibition of autophagy by *bec-1* RNAi significantly reduced the collagen levels in 8-day-old worms at 15 °C. The data are expressed as percent of control (the value of 1-day-old worms at 20 °C). These results are means ± SD (*n* = 3). *P < 0.05, 8-day-old worms with EV at 15 °C versus 8-day-old worms with EV at 20 °C, or 8-day-old worms with *bec-1* RNAi at 15 °C. ns, not significant. *P*-values (**g**) were calculated using a one-way ANOVA followed by a Student-Newman-Keuls test

We investigated tissue-specific activities of autophagy in the regulation of lifespan. Interestingly, epidermal-specific knockdown of *bec-1*, *let-512*, and *epg-1* by RNAi significantly reduced lifespan extension in worms at 15 °C (Fig. 5a–c, Supplementary Data 1). In contrast, intestinal-specific RNAi of *bec-1*, *let-512*, and *epg-1* did not impair lifespan extension (Fig. 5d–f, Supplementary Data 1).

A recent study has demonstrated that functional enhancement of collagen production and the extracellular matrix remodeling are essential signatures of longevity assurance in worms^44^. In *C. elegans*, collagens form basement membranes and the cuticle, the latter is mainly synthesized by epidermis^45^. Our observations that autophagy in the epidermis is required for long lifespan prompted us to determine whether low temperature affects the collagen content in worms. As expected, the collagen levels of worms were reduced in 8-day-old worms, compared with 1-day-old worms at both 15 and 20 °C (Fig. 5g). However, the collagen levels were significantly higher in 8-day-old worms at 15 °C than in 8-day-old worms at 20 °C. Interestingly, knockdown of *bec-1, let-512*, and *epg-1* by RNAi reduced the collagen levels in 8-day-old worms at 15 °C, but not at 20 °C (Fig. 5g, Supplementary Fig. 13a, b). These results suggest that autophagy delays the loss of collagen at low temperature, which is probably beneficial for longevity.

## Discussion

**Fig. 6:**
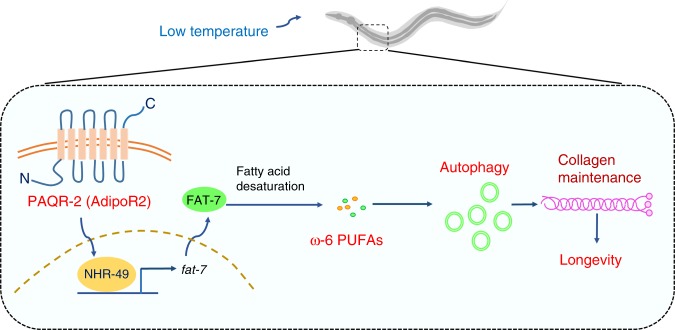
Schematic model for the PAQR-2 signaling-mediated longevity at low temperature. At low temperature, the adiponectin receptor PAQR-2 upregulates the expression of *fat-7*, which encodes a stearic CoA desaturase, by the nuclear receptor NHR-49 in worms. FAT-7 promotes the biosynthesis of two ω-6 PUFAs, γ-linolenic acid and arachidonic acid, which in turn induce autophagy. Increased autophagic activity in the epidermis delays an age-related decline in collagen contents to promote longevity

In this study, we characterized a mechanism in coupling low temperature to autophagic activity to delay chronological aging and extend lifespan in *C. elegans* (Fig. 6). Our results indicate that, in addition to TRPA-1-mediated calcium signaling^14^, the adiponectin receptor PAQR-2 signaling promotes lifespan extension by inducing autophagy in response to low temperature. Although autophagy is known to be required for long lifespan in a variety of worm genetic models^23,46^, the contribution of autophagy in individual tissues to longevity remained largely unclear. Two recent studies revealed that autophagy in the intestine played a crucial role to ensure lifespan extension in long-lived *eat-2* and *glp-1* mutants, but not in *daf-2* mutants^47,48^. In this study, we found that autophagy in the epidermis promoted lifespan extension at low temperature. These observations emphasize the differences in autophagy function among tissues in different longevity models.

Although the transcription factor HLH-30/TFEB is a known master regulator of the autophagy process^49^, our results showed that HLH-30 was not involved in autophagy and lifespan extension in worms at low temperature. Instead, our results demonstrated that the adiponectin receptor PAQR-2 signaling was involved in autophagy in worms at low temperature. *paqr-2* was originally identified to be a crucial gene for the growth of larva of *C. elegans* at 15 °C, with the *paqr-2* mutants unable to grow from L1 to fertile adult^30^. A subsequent study has demonstrated that PAQR-2 participates in low-temperature adaptation by upregulating the expression of *fat-7* via NHR-49^31^. FAT-7 in turn mediates fatty-acid desaturation, thus promoting membrane fluidity at 15 °C. Recently, Horvitz and colleagues have revealed that PAQR-2 increases intracellular levels of C11/C12 fatty acids, which can bind to and activate NHR-49 at 15 °C^42^. Our data indicated that the PAQR-2/NHR-49/FAT-7 signaling promoted autophagy in adult worms at low temperature. The upregulation of *fat-7* led to an increase in the production of GLA and AA. Furthermore, these two PUFAs were capable of fully restoring autophagy in worms subjected to genetic inactivation of *paqr-2*, *nhr-49*, and *fat-7* at low temperature. Thus, PUFAs are the downstream molecules for the PAQR-2/NHR-49/FAT-7 signaling.

In this study, we showed that besides GLA and AA, several other unsaturated fatty acids, such as OA, LA, and DGLA (but not ω-3 AA and EPA) also restored the autophagy activities in *fat-7(wa36)* mutants. A similar observation has been reported before in wild-type worms and mammalian cells, where supplementation with AA and DGLA, but not with EPA, can activate autophagy^26^. Interestingly, LA, GLA, DGLA, and AA belong to the ω-6 unsaturated fatty acids, whereas ω-3 AA and EPA are the ω-3 unsaturated fatty acids. The dysfunction of autophagy has been linked to a wide range of clinically relevant conditions, including neurodegenerative diseases, metabolic disorders, chronic inflammation, and cardiovascular disorders^50^. The beneficial impact of ω-6 unsaturated fatty acids on activating autophagy suggests that dietary supplementation with ω-6 unsaturated fatty acids could be of great help in the prevention and treatment of multiple disorders. Although OA, LA, GLA, and DGLA are metabolic precursors of AA, our results demonstrated that these fatty acids could restore autophagic activity in an AA-independent manner. Clearly, the mechanism by which ω-6 unsaturated fatty acids induce autophagy needs to be investigated further in light of the current results.

Two previous studies have demonstrated that worms overexpressing *nhr-49* and the gain-of-function *nhr-49(et7)* mutants display a long lifespan at normal temperature^51,52^. We observed that genetic inactivation of *paqr-2*, *nhr-49*, and *fat-7* significantly reduced lifespan at low temperature. However, supplementation of GLA and AA partially rescued the short lifespan in *paqr-2(tm3410)* and *nhr-49(nr2041)* mutants, and fully restored the lifespan in *fat-7(wa36)* mutants to that of wild-type worms at low temperature. Thus, the activation of the PAQR-2/NHR-49/FAT-7 signaling, an essential component of the adaptation to low temperature in larva, is involved in lifespan extension in adult worms at low temperature. It should be noted that, unlike the *fat-7(wa36)* mutation that only shortened the lifespan of worms at low temperature, genetic inactivation of *paqr-2* and *nhr-49* significantly reduced lifespan at both low and normal temperatures. These results implicate that the short-lifespan phenotype of worms with RNAi or mutations of *paqr-2* and *nhr-49* is not merely due to inhibition of autophagy. Indeed, supplementation with GLA and AA fully restored autophagy, but only partially rescued lifespan in worms with *paqr-2* and *nhr-49* mutations or RNAi at low temperature. Thus, genetic inactivation of *paqr-2* and *nhr-49* is detrimental to the fitness of worms.

Accumulating evidence suggests that the malfunctioning of autophagy is related to a wide range of age-related disorders^53^. For example, muscle-specific knockout of the autophagy gene, Atg7, causes profound muscle atrophy and age-dependent decrease in force in mice^54^. In both humans and rodents, the induction of autophagy in the skeletal muscle is an important mechanism of exercise-induced health benefits via the maintenance of muscle homeostasis^55,56^. In worms, autophagy in the intestine extends the lifespan by improving intestinal integrity and motility of *eat-2* mutants^47^. Our data demonstrated that autophagy inhibition accelerated an age-dependent loss of total collagen in worms. It is believed that collagen maintenance and extracellular matrix youthfulness are beneficial for long lifespan in worms^44^. Thus, autophagy in the epidermis slows aging by supporting collagen maintenance and extracellular matrix remodeling in worms at low temperature.

In *C. elegans*, NHR-49, a homolog of the mammalian nuclear receptor gene HNF4, functions similarly to that of peroxisome proliferator-activated receptor (PPARα)^51,57^. In mammals, adipoR2 is involved in the activation of PPAR-α signaling pathway^58,59^. Mice with reduced core body temperature exhibit long lifespan, which is independent of calorie restriction^13^. Increased serum adiponectin levels have been observed in rats kept at 4 °C for 24 h^60^ and in human subjects wearing a 10 °C liquid-conditioned suit for 2 h^61^. As adiponectin induces autophagy in a variety of tissues, such as heart^62^, liver^63^, and skeletal muscle^64^, low temperatures likely induce autophagic activity in mammals. Indeed, elevated autophagic activity is observed in multiple tissues of mice after exposure to 4 °C for 1 h^65^. Our current study thus suggests that the adiponectin/AdipoR2/PPARα signaling is a potential pathway that induces autophagy in mammals at low temperatures. Interestingly, the levels of adiponectin and AdipoR2 are also increased in blood and skeletal muscle during exercise^55,66^. Characterization of the role for this pathway may provide mechanistic insights into how exercise promotes health benefits and improves physical performance.

## Methods

### Nematode strains

Multiple mutants and transgenic strains were used in this study. Strains DA2123(*lgg-1p::gfp::lgg-1*), *trpa-1(ok999)*, the epidermal-specific RNAi strain NR222 *rde-1(ne219)V; kzIs9([pKK1260(lin-26p::nls::gfp), pKK1253(lin-26p::rde-1), pRF4 (rol-6)])* in which rde-1 expression is controlled under an epidermal-specific *lin-26* gene promoter^67^, *paqr-2(tm3410)*, *nhr-49(nr2041)*, and MAH235*(sqIs19 [hlh-30p::hlh-30::GFP* + *rol-6(su1006)])* were all kindly provided by the Caenorhabditis Genetics Center (CGC; http://www.cbs.umn.edu/CGC), funded by NIH Office of Research Infrastructure Programs (P40 OD010440). The nematode strain for intestinal-specific RNAi, MGH170(*sid-1(qt9); Is[vha-6pr::sid-1];Is[sur-5pr::GFPNLS*]), was kindly provided by Dr. Gary Ruvkun (Massachusetts General Hospital, Harvard Medical School). The strains *fat-7(wa36)*, BX113*[lin-15B&lin-15A(n765) X; fat-7::GFP* + *lin15(*+*)]* and BX115 *[lin-15B&lin-15A(n765) X; fat-6::GFP* + *lin15(*+*)]* were kindly provided by Dr. Bin Liang (Kunming Institute of Zoology, Chinese Academy of Science). Strains bpIs151 *[sqst-1p::sqst-1::GFP* + *unc-76(*+*)]* and bpIs239 *[W07G4.5p::W07G4.5::GFP* + *unc-76(*+*)]* were kindly provided by Dr. Hong Zhang (Institute of Biophysics, Chinese Academy of Science). Mutants were backcrossed three times into the N2 strain used in the laboratory. All strains were maintained on nematode growth media (NGM) and fed with *E. coli* OP50 at 20 °C^68^.

### RNA interference

RNAi bacterial strains containing targeting genes were obtained from the Ahringer RNAi library^69^. *E. coli* strain HT115(DE3) expressing dsRNA was grown overnight in LB broth containing 100 μg/ml ampicillin at 37 °C, then spread onto NGM plates containing 100 μg/ml ampicillin and 1 mM isopropyl 1-thio-β-D-galactopyranoside (IPTG). The RNAi-expressing bacteria were then grown at 25 °C overnight. Synchronized L1 larvae were placed on the plates at 20 °C until they reached maturity. Young adult animals were used for further experiments.

### Autophagy analysis

Synchronized populations of transgenic worms carrying GFP::LGG-1, SQST-1::GFP, and W07G4.5::GFP were cultivated at 20 °C until they reached the late L4 stage, and then shifted to 15, 20, and 25 °C. After 24 h of growth at different temperatures, the transgenic worms were immediately mounted in M9 onto microscope slides. Some worms expressing GFP::LGG-1 were injected with 50 mM BafA or DMSO. Two hours after injection, these worms were immediately mounted in M9 onto microscope slides. The slides were viewed using a Zeiss Axioskop 2 plus fluorescence microscope (Carl Zeiss, Jena, Germany) with a digital camera. For the transgenic worms carrying GFP::LGG-1, GFP::LGG-1 positive puncta were counted in the seam cells or the intestine. At least 30 worms were examined per assay in three independent experiments. For the transgenic worms carrying SQST-1::GFP and W07G4.5::GFP, fluorescence intensity was quantified by using the ImageJ software (NIH). Three plates of about 50 worms per plate were tested per assay and all experiments were performed three times independently.

### Lifespan analysis

All lifespan measurements were performed on NGM agar plates containing *E. coli* OP50 with the exception of those involving RNAi, in which case they were conducted on *E. coli* HT115. Synchronized populations of worms were cultivated at 20 °C until they reached the late L4 stage, and then shifted to 15, 20, and 25 °C, respectively, to score adult lifespan at each of these temperatures. The first day of adulthood was recorded as day 1. Animals were transferred to new plates every day during their reproductive period. After that, worms were transferred every third day. The number of worms was monitored every day. Worms that did not move when gently prodded and displayed no pharyngeal pumping were marked as dead.

### Age-related phenotypic marker assays

One-day-old worms were placed on RNAi food until day 8 of adulthood and the following age-related phenotypes were scored^37,44^. (1) Pharyngeal pumping was measured by counting the number of contractions in the terminal bulb of pharynx in 30 s intervals. (2) Body bending was determined by counting the number of body bends in 30 s intervals. Ten worms were examined per assay in 20 independent experiments. (3) Lipofuscin levels were determined by mounting animals onto microscope slides, and taking bright-field and 4′,6-diamidino-2-phenylindole (DAPI) channel pictures with a Zeiss Axioskop 2 Plus fluorescence microscope. Blue fluorescence from the DAPI channel pictures were analyzed using ImageJ (NIH). At least 30 worms were examined per assay in three independent experiments. (4) Pharyngeal degeneration. Images of pharynxes were scored on an ordinal scale with scores 1–5, with 1 representing a youthful appearance and 2–5 denoting increasing orders of overall deterioration in the pharynxes. The percentages of pharyngeal degeneration were calculated in total worms. For each time point, three independent experiments were carried out. In each experiment, 20–30 of worms were calculated.

### Scoring of HLH-30 nuclear accumulation

Synchronized populations of worms expressing *hlh-30p*::*hlh-30::gfp* were cultivated at 20 °C until they reached the late L4 stage, and then shifted to 15, 20, and 25 °C. After 12 and 24 h of growth at different temperatures, the worms were immediately mounted in M9 onto microscope slides. The slides were viewed using a Zeiss Axioskop 2 Plus fluorescence microscope. The status of HLH-30 localization was categorized as cytosolic localization or nuclear localization when localization was observed throughout the body from head to tail. About 100 nematodes were counted per assay and all experiments were performed three times independently.

### Western blotting

After treatment, worms were homogenized in liquid nitrogen. The homogenate was lysed on ice for 60 min in RIPA buffer (Beyotime Institute of Biotechnology, Haimen, China). Fifty micrograms of total protein lysates were loaded per well and separated on a 10% SDS polyacrylamide gel. Proteins were then transferred to immobilon-PSQ transfer PVDF membrane (Millipore, Bedford, MA). The primary antibodies were anti-GFP (#M20004, mouse mAb, 1:1000 dilution; Abmart Inc., Shanghai, China) and anti-actin antibodies (#ab14128, mouse mAb, 1:1000 dilution; Abcam, Cambridge, MA). The secondary antibodies were HRP-conjugated anti-mouse IgG (#HS201-01, 1:5000 dilution; Beijing TransGen Biotech Co., China). An imaging system (Amersham Imager 600) was used for documentation of the western blotting results. Protein intensity was analyzed using ImageJ (NIH). Uncropped scans of all western blots are placed in the Supplementary Fig. 14.

### Quantitative RT-PCR

Total RNA was extracted from worms with TRIzol Reagent (Invitrogen, Camarillo, CA). Random-primed cDNAs were generated by reverse transcription from total RNA with QuantScript RT Kit (Tiangen, Beijing, China). Quantitative real-time PCR was performed using SYBR Premix Ex Taq (Takara) on a Roche LightCycler 480 System (Roche Applied Science, Mannheim, Germany). *pan-actin* was used as an internal control. The primers used for PCR were as follows: *gfp*, 5′-GACGGGAACTACAAGACA-3′ (forward) and 5′-CTATTAACAAGGGTATCAC-3′ (reverse); *pan-actin* 5′-TCGGTATGGGAC AGAAGGAC-3′ (forward) and 5′-CATCCCAGTTGGTGACGATA-3′ (reverse).

### Microscopy

For imaging fluorescence in worms, the transgenic worms carrying *fat-6::gfp* or *fat-7::gfp* were mounted in M9 onto microscope slides. The slides were viewed using a Zeiss Axioskop 2 Plus fluorescence microscope. Averages and standard errors were calculated based on more than 100 worms per assay. The fluorescence intensity was analyzed using ImageJ (NIH).

### Measurement of the complete fatty-acid profiles by LC/MS

Synchronized populations of worms were cultivated at 20 °C until they reached the late L4 stage, and then shifted to 15, 20, and 25 °C. After 24 h of growth at different temperatures, the worms were collected. The fatty-acid profiles were determined using the following protocol. Briefly, about 1 ml of packed worms were homogenized in liquid nitrogen. The homogenate was mixed with the RIPA buffer to a final volume of 1 ml. Following centrifugation at 16,600 × *g* for 30 min at 4 °C, the supernatant was collected. Five microliters of the supernatant was used to determine total protein concentration. The remaining supernatant was freeze-dried in a VirTis freeze dryer. After the resulting powders were extracted with diethyl ether, the mixture was centrifuged at 16,600 × *g* for 30 min at 4 °C to remove the insoluble residue. After the solution was dried under N2 gas using Termovap Sample Concentrator, the sample was dissolved in 0.2 ml of methanol, analyzed by Agilent 6500 Series Q-TOF UPLC/MS System (Agilent Technologies, Santa Clara, CA). The samples were separated on a ZORBAX Eclipse Plus C18 RRHD (2.1 mm × 50 mm, 1.8 μm particles; Agilent Technologies) with gradient elution. The mobile phase consisted of solvent A (water) and solvent B (methanol), both containing 0.1% formic acid. The flow rate of the mobile phase was 300 μl/min. The gradient program was as follows: 0–3 min (75% B), 3–8 min (75–100% B), and 8–12 min (100% B). Two microliters of samples were injected into the column using an autosampler. Electrospray ionization was performed in the negative ion mode using N_2_ at a pressure of 35 psi for the nebulizer with a flow of 9 L/min and a temperature of 350 °C. The capillary was set at −3500 V. Data were analyzed by using Agilent MassHunter Workstation software. C16:0, C18:0, C16:1, C18:1 *cis*, C18:1 *trans*, C18:2 n-6, C18:3 n-6, C18:3 n-3, C20:1 n-9, C20:4 n-6, and C20:5 n-3 fatty-acid standards were purchased from Sigma Chemical Company (St. Louis, MO); C20:3 n-6 fatty-acid standard was from Abcam Company (Cambridge, MA); and C20:4 n-3 fatty-acid standard was from Toronto Research Chemicals Company (Toronto, Ontario, Canada).

### Quantitative analysis of fatty-acid composition by GC/MS

Fatty-acid composition was measured by producing fatty-acid methyl esters (FAMEs) as follows^70^. Briefly, after supplementation with OA (100 μM), LA (200 μM), GLA (500 μM), or DGLA (500 μM) for 24 h, the *fat-7(wa36)* mutant worms (~10,000) were collected and washed with M9 buffer five times. To extract fatty acids and transmethylate them, the worm pellets were incubated with 1 ml of 2.5% H_2_SO_4_ in methanol in a glass test tube at 80 °C for 1 h. Then the tube was removed from water bath and cooled to room temperature. After the addition of 0.2 ml of hexane and 1.5 ml of H_2_O, FAMEs were extracted into the hexane layer by shaking vigorously and centrifuging the tubes at low speed for 1 min. Hundred microliters of top hexane layer was carefully pipetted out and placed in a new tube for subsequent GC-MS analysis.

The FAMEs were analyzed by a Shimadzu GC/MS-QP2010 Ultra spectrometer (Shimadzu Scientific Instruments, Columbia, MD) equipped with a MS with an electron impact (EI) ion source. GC separation was performed by using an Agilent DB-WAX capillary GC column (30 m × 0.25 mm and 0.25 μm film thickness), and 2 μl of each sample was injected in split mode with a 1:11 ratio. Ultrapure helium was the carrier gas and makeup. The injection port and detector temperatures were set at 230 and 250 °C, respectively. The column temperature programming started at 120 °C held for 1 min and heated up to 190 °C at a rate of 20 °C/min. The column temperature was held for 8 min at 190 °C, and then increased to 200 °C (2 °C/min) and kept for 2 min. The column was then increased to 220 °C (2 °C/min) and kept for 3 min. The MS spectra were acquired with the EI voltage as tuning voltage. The mass range selected was 35–500 *m*/*z*. The FAMEs were identified by comparing with a chromatogram from a mixture of known standards and further confirmed with their mass spectral data. Fatty-acid abundance was expressed as the percent of total fatty acids.

### Collagen assays

Collagen levels were measured by detecting hydroxyproline using the Hydroxyproline (HYP) Content Assay Kit (BC0250, Solarbio, Beijing, China) according to the manufacturer’s instructions. Briefly, synchronized populations of worms were cultivated at 20 °C until they reached the late L4 stage, and then shifted to 15 and 20 °C to determine collagen contents. Forty to seventy milligrams of 1-day-old and 8-day-old worms were collected and washed three times with M9 buffer. Worms were homogenized in liquid nitrogen. The resulting powders were pre-digested in 0.5 ml of 6 M HCl in microwave at 110 °C for 2–6 h. Once the solution was clear, 6 M HCl was added to achieve a final volume of 0.5 ml. Collagen contents were determined using the kit according to the manufacturer’s instructions. The collagen contents were expressed as micrograms of collagen per milligram of worm wet weight.

### Statistics

These results presented in each figure are mean ± SD of three independent experiments performed in triplicate. Differences in survival rates were analyzed using the log-rank test. Differences in pharyngeal deterioration were analyzed using the Wilcoxon rank sum test. The statistical significance of differences in protein expression, the numbers of GFP::LGG-1 positive puncta, fluorescence intensity, was assessed by performing a one-way ANOVA followed by a Student-Newman-Keuls test. Data were analyzed using GraphPad Prism 7 (GraphPad Software Inc., La Jolla, CA).

### Reporting summary

Further information on research design is available in the Nature Research Reporting Summary linked to this article.

## Supplementary information

Supplementary Information

Description of Additional Supplementary Files

Supplementary Data 1

Reporting Summary

## Data Availability

The data that support the findings of this study are available from the corresponding author upon reasonable request.
