# Supplementary material for: Adiponectin receptor PAQR-2 signaling senses low temperature to promote C. elegans longevity by regulating autophagy
Source: Nat Commun. 2019 Jun 13;10:2602. doi: 10.1038/s41467-019-10475-8 (PMC6565724; doi:10.1038/s41467-019-10475-8)
Supplement: Supplementary file 1 — Supplementary Information [file 41467_2019_10475_MOESM1_ESM.pdf]

Supplementary Information

Adiponectin receptor PAQR-2 signaling senses low temperature to promote *C. elegans* longevity by regulating autophagy

Chen. *et al*

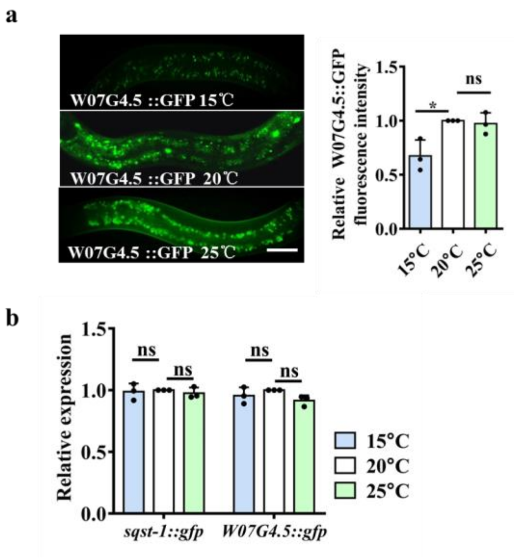

Supplementary Figure 1. The effect of low temperature on the expression of *sqst-1* and *W07G4.5* in worms. Day 1 adult worms were grown at 15°C, 20°C, and 25°C for 24 h, respectively. (a) The expression of W07G4.5::GFP in adult worms. The right panel shows quantification of GFP levels. *$P < 0.05$, 15°C versus 20°C. Scale bars: 50 μm. These results are means ± SD of three independent experiments (n = 50-55 worms per experiment). (b) The mRNA levels of *sqst-1::gfp* and *W07G4.5::gfp* in worms at 15°C, 20°C and 25°C. These results are means ± SD (n = 3). ns, not significant. *p*-Values throughout were calculated using a one-way ANOVA followed by a Student-Newman-Keuls test.

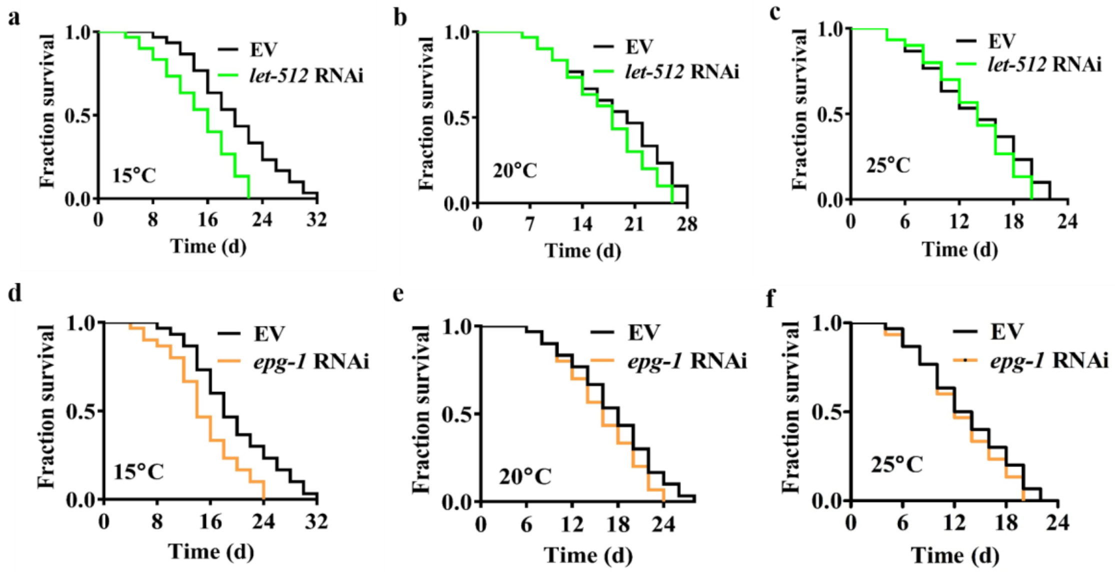

Supplementary Figure 2. Knockdown of *let-512* or *epg-1* by RNAi shortens lifespan of worms at low temperature. (a) 15°C; $P < 0.01$ versus wild type animals (N2) with empty vector (EV); (b) 20°C; (c) 25°C. Not significant, versus N2 with EV. (d) 15°C; $P < 0.01$ versus N2 with EV; (e) 20°C; (f) 25°C. Not significant, relative to N2 with EV. *p*-Values throughout were calculated using log-rank test. See Supplementary Data 1 for details.

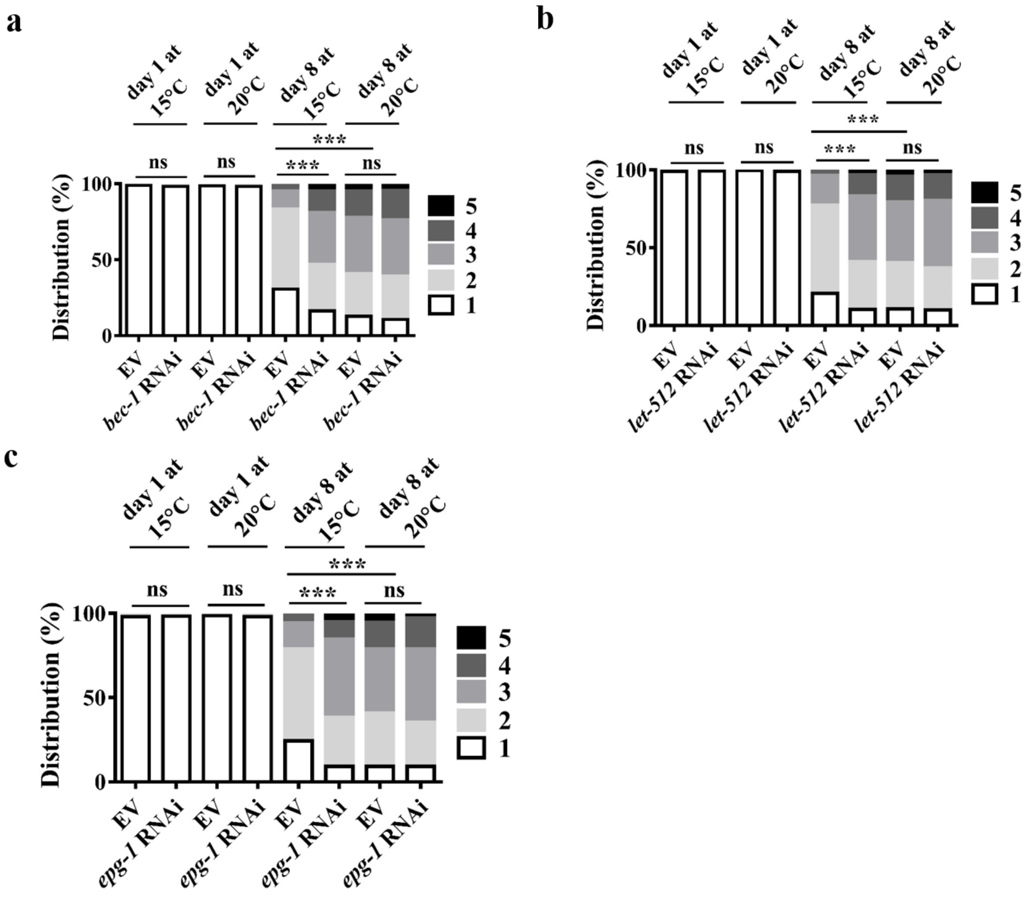

Supplementary Figure 3. Knockdown of *bec-1*, or *let-512*, or *epg-1* by RNAi aggravates pharyngeal deterioration at low temperature. (a-c) Classification of pharynx structure in adult worms subjected to *bec-1* (a), *let-512* (b), and *epg-1* (c) RNAi at days 1 and 8 at 15°C and 20°C. For each time point, three independent experiments were carried out. The percentages of pharyngeal degeneration were calculated in total worms (n = 20-30 worms each experiment). ****P*< 0.001, eight-day-old worms with empty vector (EV) at 15°C versus eight-day-old worms with EV at 20°C, or eight-day-old worms with *bec-1* RNAi at 15°C. ns, not significant. *p*-Values were calculated using the Wilcoxon rank sum test.

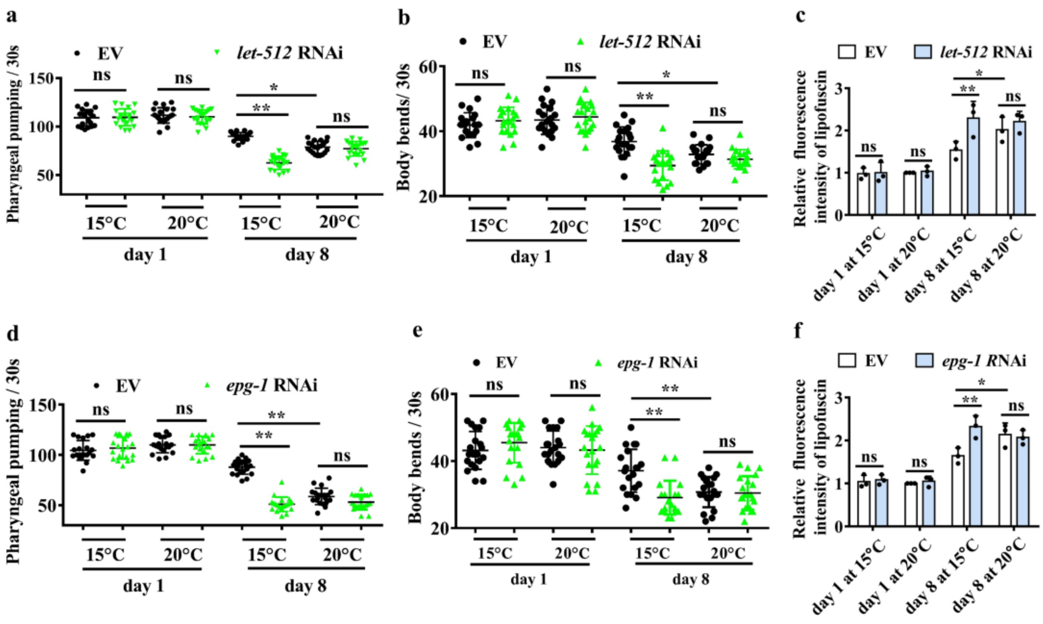

Supplementary Figure 4. Knockdown of *let-512* and *epg-1* by RNAi aggravates appearance of aging markers at low temperature. The appearance of aging markers in worms at adults subjected to *let-512* (a-c) and *epg-1* (d-f) RNAi at 15°C and 20°C. (a and d) Pharyngeal pumping; (b and e) Body bending; These results are means ± SD (n = 20). **P*< 0.05; ***P*< 0.01, eight-day-old worms with empty vector (EV) at 15°C versus eight-day-old worms with EV at 20°C; ***P*< 0.01, eight-day-old worms with EV at 15°C versus eight-day-old worms with *let-512* or *epg-1* RNAi at 15°C. ns, not significant. Ten of worms were examined per experiment. (c and f) Lipofuscin autofluorescence. These results are means ± SD of three independent experiments (n = 30-33 worms per experiment). **P*< 0.05, eight-day-old worms with EV at 15°C versus eight-day-old worms with EV at 20°C; ***P*< 0.01, eight-day-old worms with EV at 15°C versus eight-day-old worms with *let-512* or *epg-1* RNAi at 15°C. ns, not significant. *p*-Values throughout were calculated using a one-way ANOVA followed by a Student-Newman-Keuls test.

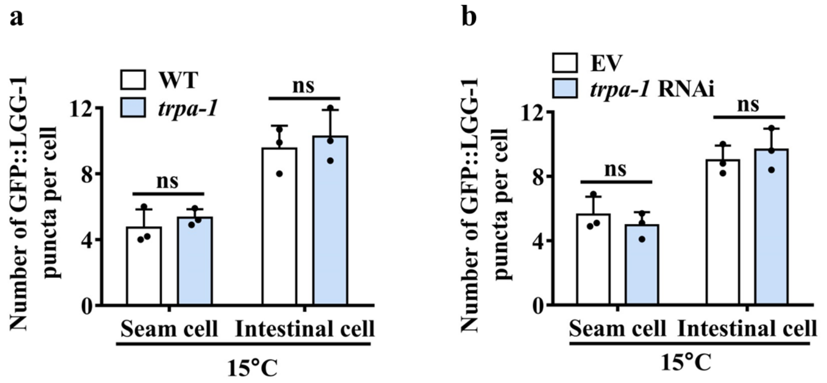

Supplementary Figure 5. *trpa-1* is not involved in the induction of autophagy at low temperature. (a) *trpa-1(ok999)* mutants. (b) *trpa-1* RNAi. These results are means ± SD of three independent experiments (n = 30-34 worms per experiment). EV, empty vector. ns, not significant. *p*-Values throughout were calculated using a one-way ANOVA followed by a Student-Newman-Keuls test.

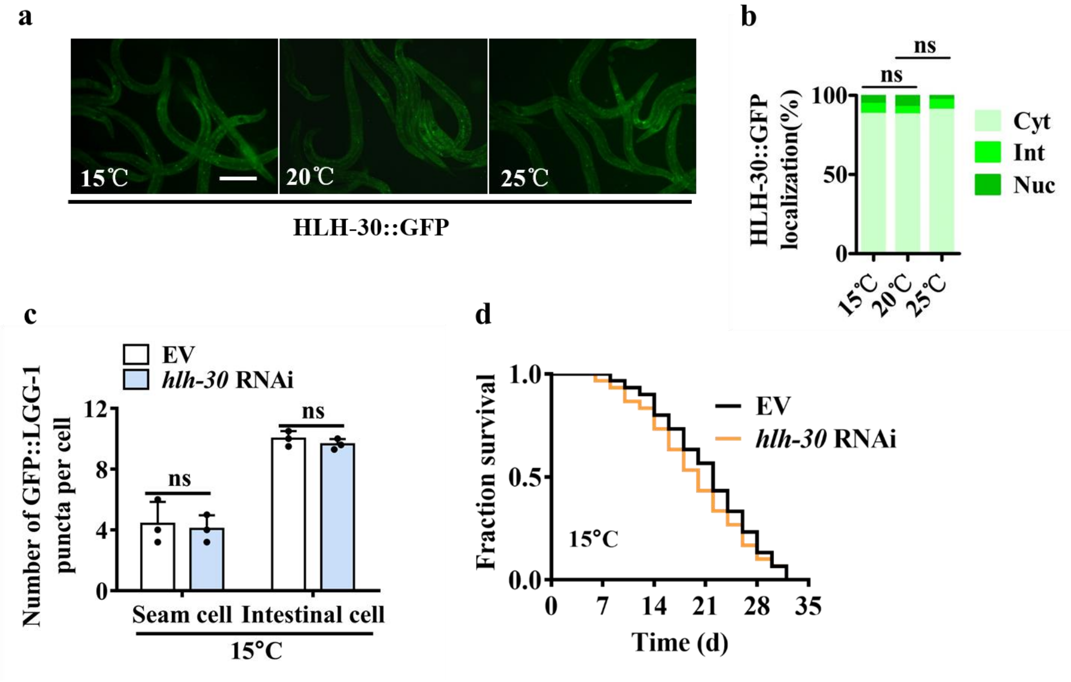

Supplementary Figure 6. ***hlh-30*** **is not involved in lifespan extension in worms at low temperature.** (a) HLH-30 nuclear localization did not occur in worms at 15°C. Scale bars: 150 μm. (b) Quantification of HLH-30 distribution. For each time point, three independent experiments were carried out. The percentages of HLH-30 distribution were calculated in total worms (n = 100-109 worms each experiment). ns, not significant (Wilcoxon rank sum test). (c) Knockdown of *hlh-30* by RNAi did not influence autophagy in worms at 15°C. These results are means ± SD of three independent experiments (n = 30-33 worms per experiment). ns, not significant (One-way ANOVA followed by a Student-Newman-Keuls test). EV, empty vector. (d) Knockdown of *hlh-30* by RNAi did not influence lifespan in worms at 15°C. (Log-rank test, not significant, *hlh-30* RNAi versus animals with EV). See Supplementary Data 1 for details.

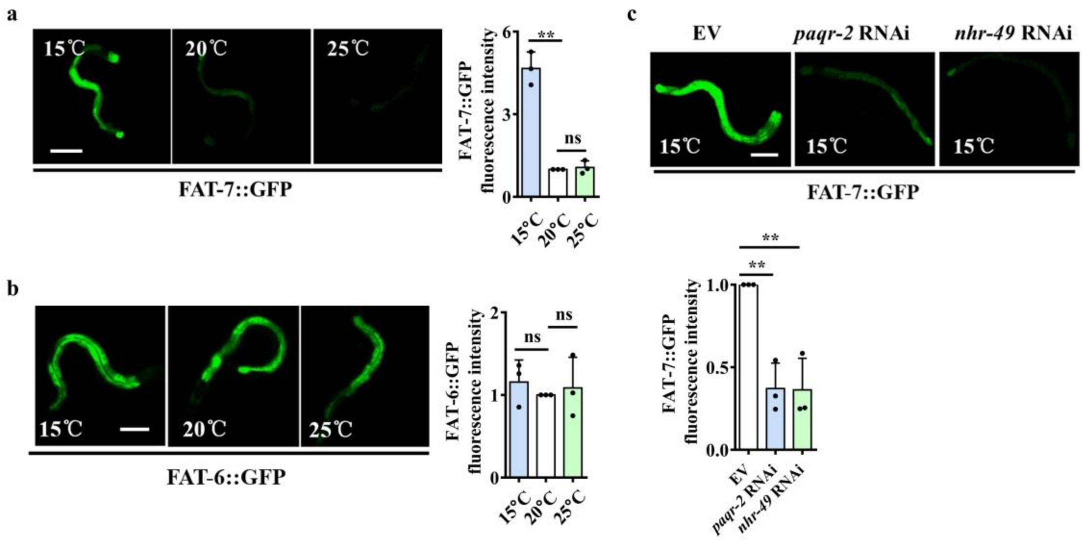

Supplementary Figure 7. Up-regulation of *fat-7* is mediated by PAQR-2 and NHR-49 at low temperature. (a) The expression of *fat-7::gfp* (a) was up-regulated in worms at 15 ℃, The right panel shows quantification of GFP levels. $^{**}P < 0.01$, 15°C versus 20°C. ns, not significant. (b) The expression of *fat-6::gfp* was not altered. The right panel shows quantification of GFP levels. ns, not significant. (c) Knockdown of *paqr-2* and *nhr-49* by RNAi led to a decrease in the expression of *fat-7::gfp* in worm at 15 ℃. The lower panel shows quantification of GFP levels. $^{**}P < 0.01$, worms with EV versus *paqr-2* or *nhr-49* RNAi at 15°C. These results are means ± SD of three independent experiments (n = 100-106 worms per experiment). *p*-Values throughout were calculated using a one-way ANOVA followed by a Student-Newman-Keuls test. Scale bars: 200 μm.

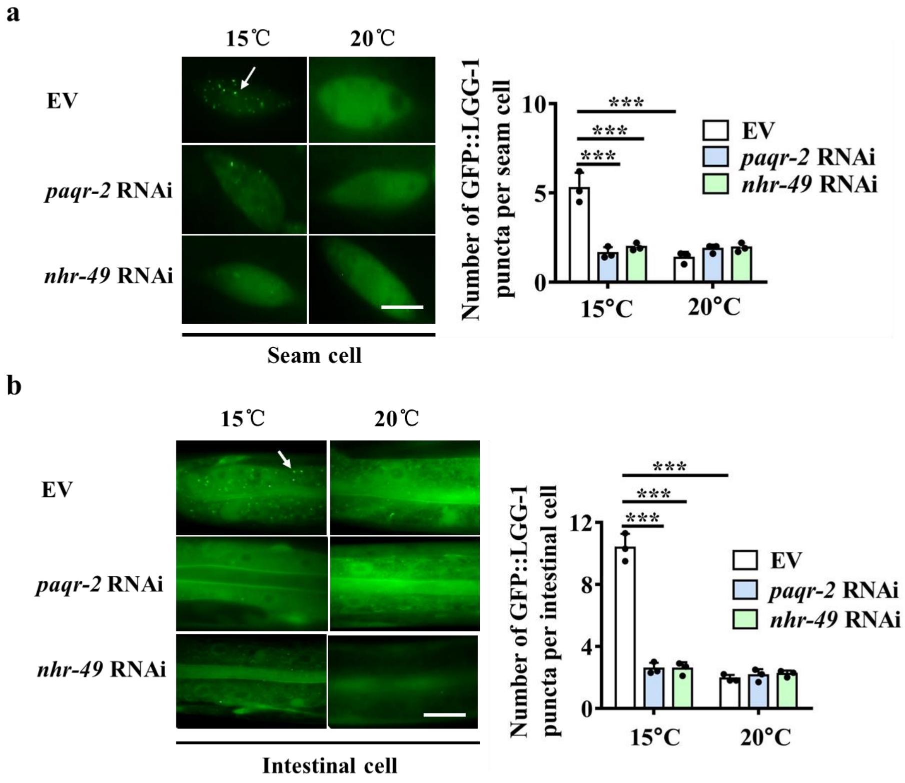

Supplementary Figure 8. Knockdown of *paqr-2* and *nhr-49* by RNAi suppresses autophagy at low temperature. (a and b) Representative images of autophagosomes (GFP::LGG-1 puncta) in the seam cells (a) and intestinal cells (b) of worms subjected to *paqr-2* and *nhr-49* RNAi at 15°C and 20°C, respectively. The arrow denotes a representative autophagosome. The numbers of GFP::LGG-1 puncta were counted in the seam cells (a, right panel) and intestinal cells (b, right panel). These results are means ± SD of three independent experiments (n = 30-32 worms per experiment). *** $P< 0.001$, worms subjected to RNAi versus those with empty vector (EV) at 15°C or worms with EV at 15°C versus worms with EV at 20°C. *p*-Values throughout were calculated using a one-way ANOVA followed by a Student-Newman-Keuls test. Scale bars: seam cells, 10 μm; intestinal cells, 20 μm.

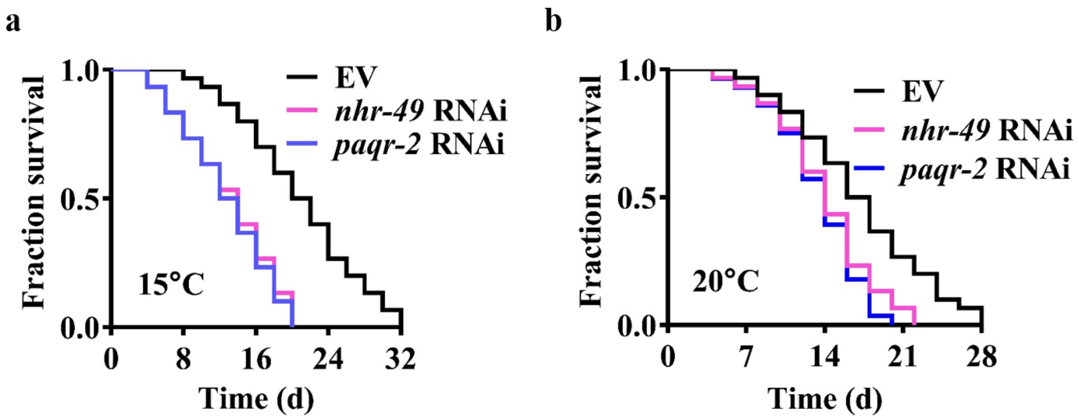

Supplementary Figure 9. Knockdown of *paqr-2* and *nhr-49* by RNAi shortens lifespan of worms at 15°C and 20°C. (**a**) 15°C. $P < 0.001$ versus empty vector (EV). (**b**) 20°C. $P < 0.01$ versus EV. *p*-Values were calculated using log-rank test. See Supplementary Data 1 for details.

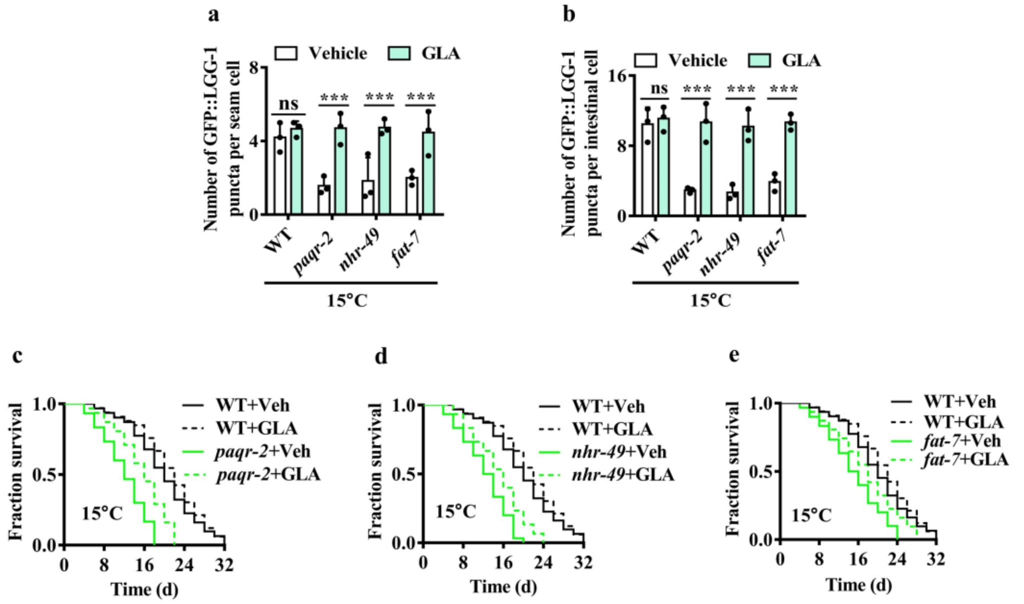

Supplementary Figure 10. γ-Linolenic acid supplementation restores the activation of autophagy and rescues the short lifespan in *paqr-2(tm3410)*, *nhr-49(nr2041)*, and *fat-7(wa36)* mutants at 15°C. (a and b) Supplementation with γ-linolenic acid (GLA, 500 μM) significantly restored autophagy in both seam cells (a) and intestinal cells (b) in *paqr-2(tm3410)*, *nhr-49(nr2041)*, and *fat-7(wa36)* mutants at 15°C. These results are means ± SD of three independent experiments (n = 30-33 worms per experiment). *** $P< 0.001$ versus vehicle. *p*-Values (a and b) were calculated using a one-way ANOVA followed by a Student-Newman-Keuls test. (c and d) Supplementation with GLA (500 μM) partially restored lifespan in *paqr-2(tm3410)* (c) ($P< 0.001$ versus vehicle) and *nhr-49(nr2041)* (d) ($P< 0.01$ versus vehicle) at 15°C. Veh, vehicle. (e) Supplementation with GLA (500 μM) fully rescued lifespan in *fat-7(wa36)* mutant worms at 15°C ($P< 0.05$ versus vehicle). *p*-Values (c-e) were calculated using log-rank test. See Supplementary Data 1 for details.

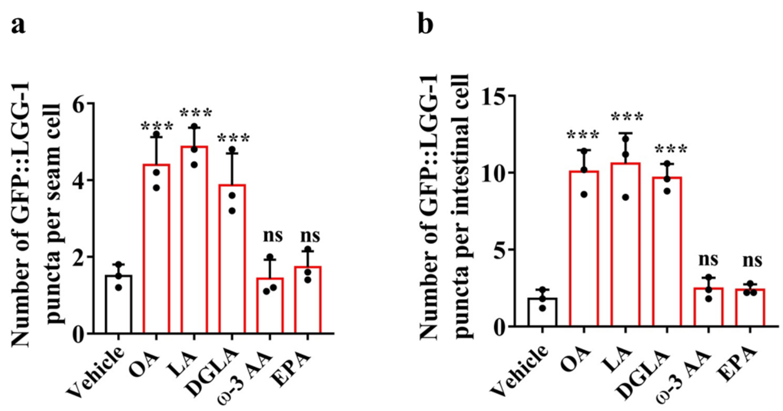

Supplementary Figure 11. The effect of several unsaturated fatty acids on autophagy in *fat-7(wa36)* mutants at 15°C. (a and b) Supplementation with oleic acid (OA, 100 μM), or linoleic acid (LA, 200 μM), or dihomo-γ-linolenic acid (DGLA, 500 μM), but not ω-3 arachidonic acid (ω-3 AA, 500 μM), or eicosapentaenoic acid (EPA, 500 μM), significantly restored autophagy in both seam cells (a) and intestinal cells (b) in *fat-7(wa36)* mutants at 15°C. These results are means ± SD of three independent experiments (n = 30-34 worms per experiment). *** $P < 0.001$ versus vehicle. ns, not significant. *p*-Values throughout were calculated using a one-way ANOVA followed by a Student-Newman-Keuls test.

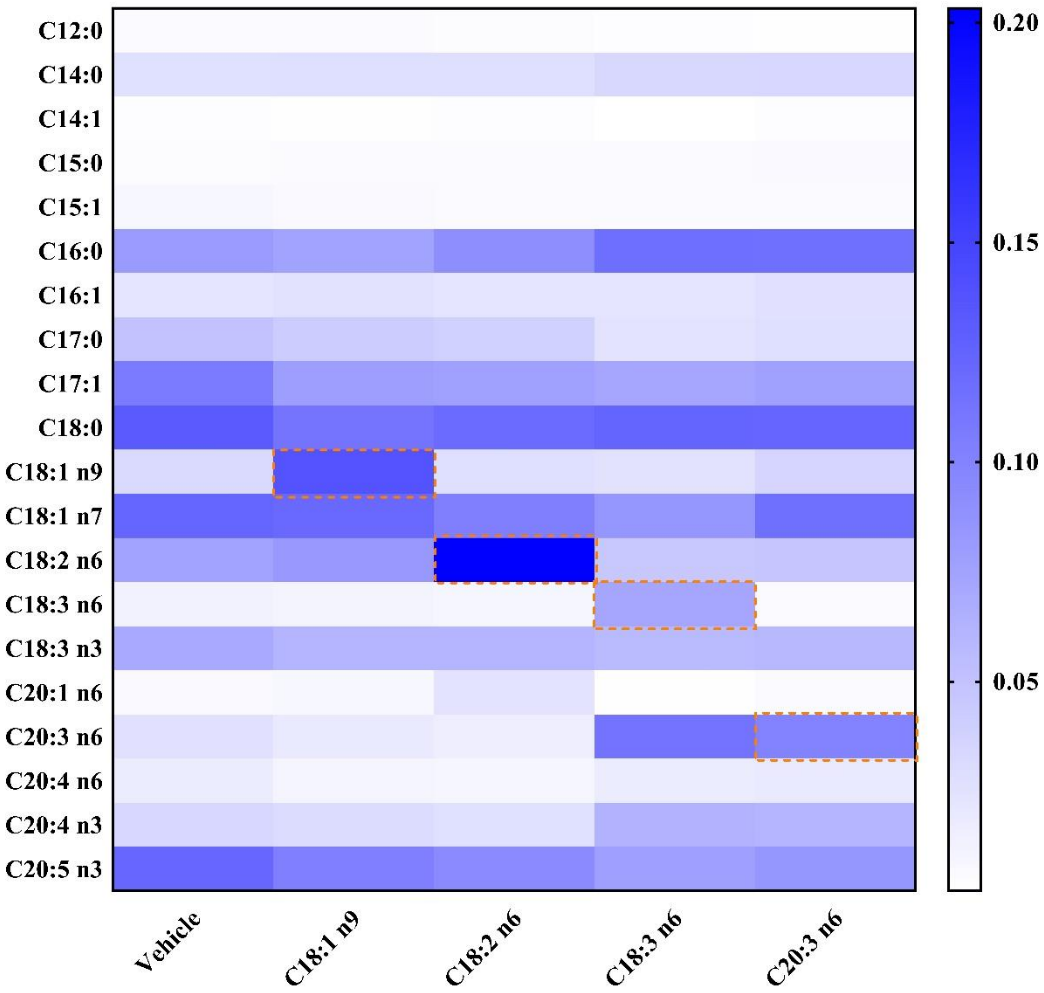

Supplementary Figure 12. Effect of fatty acid supplementation on fatty acid composition in *fat-7(wa36) mutants* at 15°C. The fatty acid composition was detected by GC/MS after supplementation with oleic acid (OA, C18:1n9, 100 μM), linoleic acid (LA, C18:2n6, 200 μM), γ-linolenic acid (GLA, C18:3n6, 500 μM), or dihomo-γ-linolenic acid (DGLA, C20:3n6, 500 μM). Each fatty acid was expressed as a percentage of the total fatty acids.

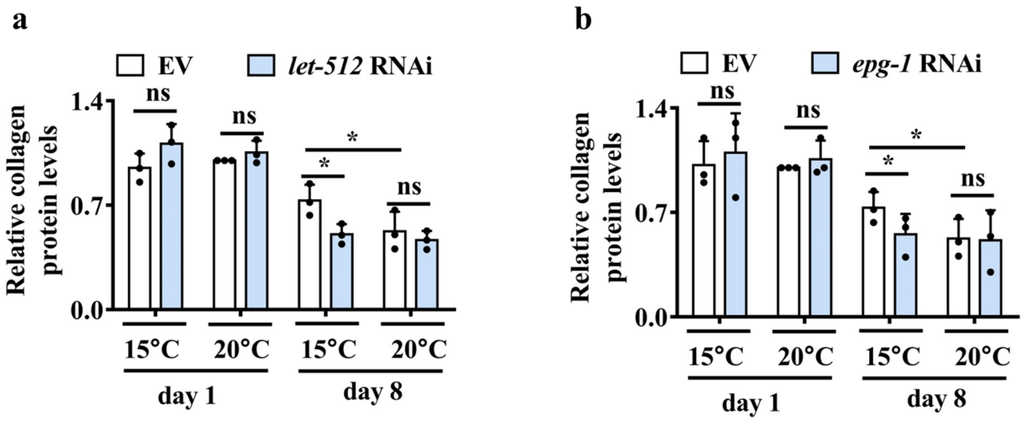

Supplementary Figure 13. Knockdown of *let-512* and *epg-1* by RNAi significantly reduces the collagen levels in eight-day-old worms at 15°C. (a and b) The relative collagen levels in worms. The data are expressed as percent of control (the value of one-day-old worms at 20°C). (a) *let-512* RNAi; (b) *epg-1* RNAi. EV, empty vector. *$P < 0.05$, eight-day-old worms with EV at 15°C versus eight-day-old worms with EV at 20°C, or eight-day-old worms with *let-512* and *epg-1* RNAi at 15°C. ns, not significant. These results are means ± SD (n = 3). *p*-Values throughout were calculated using a one-way ANOVA followed by a Student-Newman-Keuls test.

Fig.1b

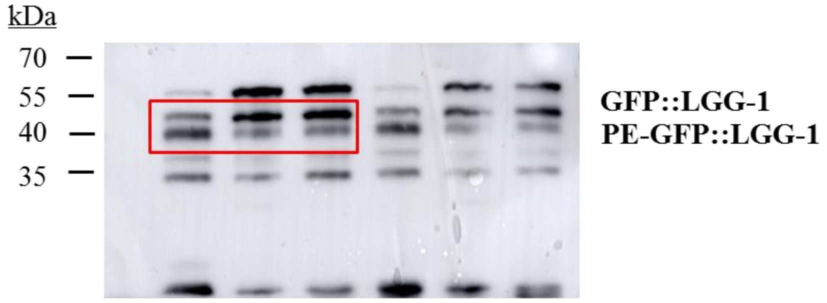

Fig.1f

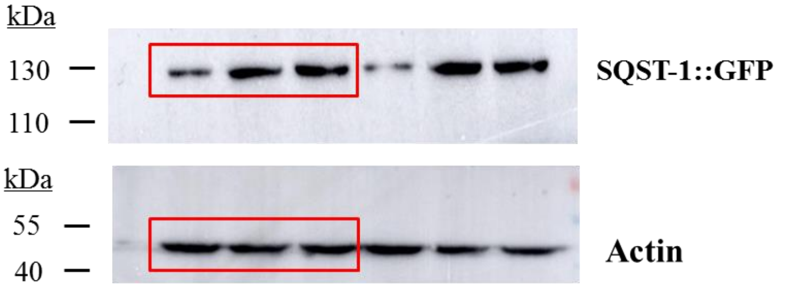

Supplementary Figure 14. Full scans for Figure 1b and f.

Supplementary Table 1 The percentages of the total lifespan of *C. elegans* strains

Strain	Temp	Max lifespan	Fraction of lifespan		Figure in the text
			1th day	8th day	
WT+EV	15℃	32	3.13%	25.00%	Fig.2d-f<br>Fig.S3a-c<br>Fig.S4a-f<br>Fig.5g<br>Fig.S13a,b
	20℃	28	3.57%	28.57%	
bec-1 RNAi	15℃	26	3.85%	30.77%	
	20℃	26	3.85%	30.77%	
let-512 RNAi	15℃	22	4.55%	36.36%	
	20℃	26	3.85%	30.77%	
epg-1 RNAi	15℃	24	4.17%	33.33%	
	20℃	28	3.57%	25.57%	
