# Supplementary material for: Adiponectin receptor PAQR-2 signaling senses low temperature to promote C. elegans longevity by regulating autophagy
Source: Nat Commun. 2019 Jun 13;10:2602. doi: 10.1038/s41467-019-10475-8 (PMC6565724; doi:10.1038/s41467-019-10475-8)
Supplement: Supplementary file 2 — Description of Additional Supplementary Files [file 41467_2019_10475_MOESM2_ESM.pdf]

Description of Additional Supplementary Files

File Name: Supplementary Data 1

Description: Summary of lifespan experiments with *C. elegans* strains.
